# Sexuality and the Quality of Life in Older People: A Correlational Study

**DOI:** 10.1192/j.eurpsy.2022.1668

**Published:** 2022-09-01

**Authors:** N.E. Boyacıoğlu, F. Oflaz, A. Yıldız Karaahmet, B.K. Hodaeı, Y. Afşin

**Affiliations:** 1 İstanbul Üniversitesi-Cerrahpaşa Health Science Faculty, Gerontology, Büyükçekmece Yerleşkesi Alkent Mah. Yiğittürk Cad. No:/, Turkey; 2 Koç University, School Of Nursıng Faculty, Istanbul, Turkey; 3 Halic University School of Health Sciences, Midwifery, Istanbul, Turkey; 4 Istanbul University- Cerrahpasa, Midwifery, Istanbul, Turkey; 5 Mimar Sinan Fine Arts University, Institute Of Science, Statistics Master Program, Istanbul, Turkey

**Keywords:** Quality of Life, Older people, health status, sexuality

## Abstract

**Introduction:**

Sexuality, which is an essential part of human life, is an instinct with the potential to cause or be caused by health problems. Although qualitative and quantitative characteristics of sexual life evolves over time, it may continue until the age of eighties.

**Objectives:**

This descriptive-correlational study aimed to analyze the relationship between general health status, quality of life and sexual life among senior people.

**Methods:**

Study was conducted with the participation of 323 (169 female and 154 male) older people at the age of 65+. The participants were clients of the inpatient and outpatient services in a general hospital in Istanbul. General Health Questionnaire, Arizona Sexual Experiences Scale and Quality of Life Scale in Older People were used to collect data via online survey.

**Results:**

The quality of life was better and sexual problems were lower for the participants who had a partner, higher education level, lower age, a regular job, sufficient income, no chronic disease, who defined their relationship as sufficient, frequently engaged in sexual activity and who considered themselves as attractive.

**Conclusions:**

Sexuality in older people changes over time and continues to hold its importance. Researchers should consider the importance of the quality of life on sexual satisfaction in older people.

**Disclosure:**

No significant relationships.

